# Prevalence of Histoplasmosis among Persons with Advanced HIV Disease, Nigeria

**DOI:** 10.3201/eid2811.220542

**Published:** 2022-11

**Authors:** Rita O. Oladele, Iriagbonse I. Osaigbovo, Alani S. Akanmu, Olukemi A. Adekanmbi, Bassey E. Ekeng, Yahaya Mohammed, Mary A. Alex-Wele, Mark O. Okolo, Stephen T. Ayanbeku, Uchechukwu S. Unigwe, Iorhen E. Akase, Alali Dan-Jumbo, Dennis Isralski, David W. Denning, Alessandro C. Pasqualotto, Tom Chiller

**Affiliations:** Lagos University Teaching Hospital, Lagos, Nigeria (R.O. Oladele, I.E. Akase);; University of Benin Teaching Hospital, Benin City, Nigeria (I.I. Osaigbovo);; University of Lagos, Lagos, Nigeria (R.O. Oladele, A.S. Akanmu);; University of Ibadan, Ibadan, Nigeria (O.A. Adekanmbi);; University of Calabar Teaching Hospital, Calabar, Nigeria (B.E. Ekeng);; Usmanu Danfodiyo University College of Health Sciences, Sokoto, Nigeria (Y. Mohammed);; University of Port Harcourt Teaching Hospital, Port Harcourt, Nigeria (M.A. Alex-Wele); University of Jos, Jos, Nigeria (M.O. Okolo);; Federal Medical Centre, Bida, Nigeria (S.T. Ayanbeku);; University of Nigeria Teaching Hospital, Enugu, Nigeria (U.S. Unigwe);; Rivers State University Teaching Hospital, Port Harcourt (A. Dan-Jumbo);; Gilead Sciences International Medical Affairs, Global Patient Solution, San Francisco, California, USA (D. Isralski);; University of Manchester, Manchester, UK (D.W. Denning);; Universidade Federal de Ciencias da Saude de Porto Alegre, Porto Alegre, Brazil (A.C. Pasqualotto);; Centers for Disease Control and Prevention, Atlanta, Georgia, USA (T. Chiller)

**Keywords:** HIV/AIDS and other retroviruses, histoplasmosis, histoplasma, fungi, tuberculosis and other mycobacteria, infections, respiratory infections, Nigeria

## Abstract

We sought to determine the prevalence of probable disseminated histoplasmosis among advanced HIV disease (AHD) patients in Nigeria. We conducted a cross-sectional study in 10 sites across 5 of 6 geopolitical zones in Nigeria. We identified patients with urinary samples containing CD4 cell counts <200 cells/mm^3^ or World Health Organization stage 3 or 4 disease who also had >2 clinical features of disseminated histoplasmosis, and we tested them for *Histoplasma* antigen using a *Histoplasma* enzyme immune assay. Of 988 participants we recruited, 76 (7.7%) were antigen-positive. The 76 *Histoplasma* antigen–positive participants had significantly lower (p = 0.03) CD4 counts; 9 (11.8%) were also co-infected with tuberculosis. Most antigen-positive participants (50/76; 65.8%; p = 0.015) had previously received antiretroviral treatment; 26/76 (34.2%) had not. Because histoplasmosis is often a hidden disease among AHD patients in Nigeria, *Histoplasma* antigen testing should be required in the AHD package of care.

Histoplasmosis, an invasive fungal infection endemic in the Americas, Africa, and Asia, with a few cases reported among immigrants to Europe, was classified as an AIDS-defining disease in 1987 (*1*,*2*). Incidence of disseminated histoplasmosis is 5%–25% in persons with advanced HIV disease (AHD; World Health Organization [WHO]–preferred term for AIDS), and according to recent data from South America, mortality rates are similar to those for tuberculosis among this patient group (*3*,*4*). In Latin America, high prevalence rates have been reported for disseminated histoplasmosis in AHD populations in Brazil (22%; 123/570) and Mexico (30%; 85/288) (*5*,*6*). Histoplasmosis is the most common AIDS-defining infection in Guatemala, more common than tuberculosis (*7*). In a recent study from Cameroon, 26% (36/138) of HIV patients had *Histoplasma* antigen in their urine regardless of CD4 count; a 2015 report indicated a 13% (7/56) prevalence in the AHD population (*8*,*9*).

WHO in 2020 published its first guidelines for disseminated histoplasmosis among persons with AHD, including recommendations for diagnosis (*10*). WHO and the US President’s Emergency Plan for AIDS Relief (PEPFAR) recommend providing differentiated care tailored to the unique needs of different HIV patient populations. Screening, treatment, and prophylaxis for major opportunistic infections is recommended for AHD (*10*). These key evidence-based interventions reduce illness and death among this clinically unstable population. Nigeria recently adopted a package of care for AHD that includes histoplasmosis screening, which has yet to be implemented. Nigeria has the 7th highest global tuberculosis rate and, because histoplasmosis is commonly misdiagnosed as tuberculosis (*11*), the histoplasmosis rate in Nigeria is likely higher than currently estimated. A recent review also revealed that Nigeria had 124 documented historical cases of histoplasmosis, the highest number in Africa, but almost all were described before the HIV pandemic began (*12*). Therefore, the effect of histoplasmosis on AHD in Nigeria is largely unknown. Our primary objective was to determine the prevalence of histoplasmosis among AHD patients in Nigeria and to generate data that will help with designing and implementing guidelines for differentiated care.

## Methods

We conducted a cross-sectional survey in 10 sites across large areas of Nigeria during November 2019–June 2021. The geopolitical zones we captured were South East (site: Enugu), South West (Lagos and Ibadan), South South (Benin, Port Harcourt, and Calabar), North Central (Bida, Jos, and Makurdi), and North West (Sokoto). Because of insurgent activities and security challenges, North East was excluded. We included antiretroviral treatment (ART) clinics and infectious disease units, in partnership with the AIDS Prevention Initiative in Nigeria program and other implementing partners in the zones; all sites included tertiary facilities (teaching hospitals). On the basis of data from the national database, selected sites all had >30% AHD prevalence among their overall populations. Five of the sites—Ibadan, Port Harcourt, Enugu, Jos, and Calabar—had histoplasmosis cases reported before the HIV epidemic (*12*); in 1 city, Benin, *Histoplasma* exposure had recently been determined by positive histoplasmin skin tests (*13*). The other 4 sites had no documented cases of histoplasmosis.

We obtained ethics clearance from national and institutional ethics committees before recruiting participants and received permission to contact patients from principal investigators or coordinators of the ART programs at each site. Managing clinicians assisted in recruiting participants. We obtained informed written consent from each study participant after adequately explaining the study and its objectives.

We recruited both ART-naive and ART-exposed outpatient or hospitalized HIV-infected patients who had a CD4 count <200 cells/mm^3^ and met other inclusion criteria. Inclusion criteria were presence of AHD and >2 of 6 features commonly seen in patients with disseminated histoplasmosis: fever, chronic cough, weight loss, cutaneous lesions, oral ulcers, and diarrhea. Among participants in 1 study, 93.8% had fever, 87% weight loss, 76% cough; and 53.4% diarrhea (*14*). Whenever possible, we collected 2 urine samples from each participant with an interval of 1 week between collections. We collected other relevant biologic samples (sputum, bronchoalveolar lavage, skin lesion biopsy, and whole blood specimen) and stored them at −80°C for future research, including histologic and genomic studies.

### Case Definitions

For our study we used the WHO AHD definition of CD4 cell count <200 cells/mm^3^ or WHO stage 3 or 4 disease in adults and adolescents (*15*). We followed the European Organisation for Research and Treatment of Cancer Mycoses Study Group consensus definition for probable disseminated histoplasmosis as a *Histoplasma* antigenuria–positive test in the presence of compatible clinical findings (*16*).

### Data Gathering and Response

We interviewed participants and reviewed their medical records and charts using a standardized checklist. This checklist encompassed sociodemographic characteristics, signs and symptoms, occupational history or exposure (e.g., gardening, civil construction, agriculture), recreational and travel history (e.g., visits to caves or farms, travel to South America), physical examination findings, working diagnoses (including the presence of other opportunistic infections), laboratory and imaging investigations, and current medications (including ARTs).

We collected participant urine samples in sterile universal screw cap containers and transported them with ice packs in refrigerator bags. Specimens were batched and stored for <2 months at −20°C before being shipped to a central laboratory for sample processing. We tested for urine *Histoplasma* antigen using the Clarus IMMY *Histoplasma* GM Enzyme immune assay from Immuno-Mycologics (https://www.immy.com) according to manufacturer instructions. We used the 9 standard positive control range and set an optical density cutoff value of 2.0 on the basis of a 4-parameter graph. We collected sputum samples from participants suspected of having tuberculosis because of signs or symptoms, such as cough, weight loss, fever, or other suggestive syndromes, and tested the samples for tuberculosis using the Cepheid Xpert MTB/RIF assay (https://www.cepheid.com).

We communicated positive *Histoplasma* antigenuria results to managing clinicians and advised them to manage those patients with a probable diagnosis of disseminated histoplasmosis according to current standard-of-care guidelines. We contacted positive participants who had been hospitalized when recruited but released by the time testing results were received to schedule an outpatient clinic visit to propose a treatment plan. Duration of follow-up varied among sites; the longest recorded follow-up duration was 30 days for a study participant receiving antifungal therapy for treatment of histoplasmosis. Treatment, intravenous amphotericin B deoxycholate for 2 weeks followed by oral itraconazole until adequate immune reconstitution occurred, was rarely given because of logistic and financial constraints. Patients were provided ART according to national treatment guidelines.

### Data Analysis

We entered all clinical and laboratory results into a spreadsheet and subsequently analyzed data by using SPSS Statistics 21 (https://www.ibm.com). We used descriptive statistics to summarize the data and determine mean, SD, median, interquartile range [IQR], and minimum and maximum for continuous variables. We determined absolute and relative frequencies to summarize categorical variables and used χ^2^ testing to check for associations and either a 2-sample or paired-sample t-test to compare continuous variables. We stratified results by ART status (naive, experienced, failed treatment), demographics, and clinical features. We used p<0.05 as the cutoff for significant associations. 

## Results

### Sociodemographic and Clinical Data

We recruited 988 participants, 377 (38.2%) male and 611 (61.8%) female, across 10 sites (Table 1); 685 (69.3%) were outpatients, 303 (30.7%) hospitalized. All participants had clinical symptoms suggestive of tuberculosis or histoplasmosis as stipulated in the inclusion criteria. Median age was 39 years (IQR 32–47 years). The most common age range for study participants was 25–40 years (n = 484; 48.9%); 80 (8.1%) were <25 years of age, 16 (1.6%) of those 13–19 years of age, and 43 (4.4%) were >60 years of age. Among participants, 259 (26.3%) had completed tertiary education and 216 (22%) had no formal education. We classified occupations into 6 groups (Table 1); the largest proportion (n = 437; 44.2%) were professionals, followed by unskilled laborers (n = 320; 32.4%), with pensioners (n = 13; 1.3%) the least common.

**Table 1:** Sociodemographic data for study of prevalence of histoplasmosis among persons with AIDS, Nigeria

Variable	No. (%) participants	No. histoplasmosis urine Ag+/total no. (%)	p value
Geopolitical zones			0.097*
North Central	355 (35.9)	20/355 (5.6)	
North West	100 (10.1)	6/100 (6.0)	
South East	44 (4.5)	3/44 (6.8)	
South South	303 (30.7)	23/303 (7.6)	
South West	186 (18.8)	24/186 (12.9)	
Sex			0.461†
F	611 (61.8)	44/611 (7.2)	
M	377 (38.2)	32/377 (8.5)	
Age, y			0.891†
<25	80 (8.1)	5/80 (6.2)	
25–40	484 (48.9)	31/484 (6.4)	
41–60	381 (38.6)	39/381 (10.2)	
>60	43 (4.4)	1/43 (2.3)	
Educational qualification			0.920†
None	216 (22)	19/216 (8.8)	
Primary	126 (12.6)	11/126 (8.7)	
Quranic school	33 (3.3)	2/33 (6.1)	
Secondary	354 (35.8)	25/354 (7.1)	
Tertiary	259 (26.3)	19/259 (7.3)	
Occupation			<0.001†
Artisan	45 (4.6)	5/45 (11.1)	
Pensioner	13 (1.3)	1/13 (7.7)	
Professional	437 (44.2)	28/437 (6.4)	
Student	64 (6.5)	6/64 (9.4)	
Unemployed	109 (11.0)	3/109 (2.8)	
Unskilled labor	320 (32.4)	33/320 (10.3)	

*Fisher exact test. †Pearson χ^2^ test.

### Histoplasmosis and Study Outcomes

We found 76 participants had *Histoplasma* antigenuria, a 7.7% prevalence of probable disseminated histoplasmosis; 44 (57.9%) were female and 32 (42.1%) were male. Among the 76 positive cases, both the first and second samples were positive in 45 (59.2%); 6 (7.9%) participants whose first samples were positive never returned to have a second test. Most (51.3%) participants with probable disseminated histoplasmosis were in the 41–60-year age range; 47.3% were <40 years of age (Table 1). The South West zone of Nigeria had the highest rate of probable histoplasmosis (12.9%), while the North Central had the lowest prevalence (5.6%). Across the various study sites, Ibadan in the South West zone had the highest rate, 20%; Benin in the South South had the lowest prevalence, 1.4% (Appendix Figure). Among possible risk factors, only occupation (p<0.001; Table 1) and smoking (p = 0.037; Table 2) were significantly associated with histoplasmosis.

**Table 2:** Associated risk factors for histoplasmosis in study of prevalence of histoplasmosis among persons with AIDS, Nigeria

Risk factors	No. (%) participants	No. histoplasmosis urine Ag+/total no. (%)	p value
Thatched roof house			0.49*
N	917 (92.8)	72 (7.9)	
Y	71 (7.2)	4 (5.6)	
Corrugated roof house			0.379*
N	277 (28.0)	17 (6.1)	
Y	711 (72.0)	59 (8.3)	
Poultry within or around residence			0.423*
N	715 (72.4)	59 (8.3)	
Y	273 (27.6)	17 (6.2)	
Warehouse (home/place of work)			0.233†
N	915 (92.6)	73 (8.0)	
Y	73 (7.4)	3 (4.1)	
Home or place of work in forested regions			0.414†
N	825 (83.5)	66 (8)	
Y	163 (16.5)	10 (6.1)	
Home or work close to a sawmill			0.384*
N	926 (93.7)	73 (7.9)	
Y	62 (6.3)	3 (4.8)	
Contact with hunters			0.280*
N	919 (93.0)	73 (7.9)	
Y	69 (7.0)	3 (4.3)	
Recent travel to areas with caves			0.249†
N	964 (97.6)	76 (7.9)	
Y	24 (2.4)	0	
Heavy construction sites near workplace or home			0.548*
N	830 (84.0)	62 (7.5)	
Y	158 (16.0)	14 (8.9)	
Home near orchards			0.264†
N	913 (92.4)	73 (8.0)	
Y	75 (7.6)	3 (4.0)	
Smoking			0.037*
N	926 (93.7)	67 (7.2)	
Y	62 (6.3)	9 (14.5)	

*Pearson χ^2^ test. †Fisher exact test.

Probable disseminated histoplasmosis participants had significantly lower CD4 counts (p = 0.03), and almost half, 36/76 (47.4%), had been hospitalized and had a median CD4 count of 96 cells/mm3 (IQR 40.75–176.00 cells/mm3) compared with nonhistoplasmosis participants, 128 cells/mm3 (IQR 70–180 cells/mm3) (Table 3). Prevalence of probable histoplasmosis was not significantly higher among hospitalized participants, 30/303 (9.9%), than outpatients, 46/685 (6.7%; p = 0.505) Conversely, the association between tuberculosis and participant group was significant (p<0.001); hospitalized patients (59/303, 21.6%) tested positive for tuberculosis more frequently than did outpatients (58/685, 8.5%). Fifty (65.8%) participants with probable histoplasmosis were ART experienced (p = 0.015), whereas the other 26 (34.2%) were ART naive.

**Table 3:** Distribution of CD4 count cells among patients with histoplasmosis and tuberculosis in study of prevalence of histoplasmosis among persons with AIDS, Nigeria

CD4 count	No. (%) participants	No. histoplasmosis urine Ag+/total no. (%)*	No. with tuberculosi/total no. (%)†
0–50	129 (13.1)	15/129 (11.6)	11/129 (8.5)
51–100	136 (13.8)	8/136 (5.9)	36/136 (26.5)
101–200	420 (42.5)	23/420 (5.5)	11/420 (2.6)
WHO clinical stage 3/4	303 (30.7)	30/303 (9.9)	59/303 (19.5)

*p = 0.063 (Pearson χ^2^ test). †p<0.001 (Pearson χ^2^ test).

Most (788, 79.8%) study participants experienced weight loss, among whom 60 (7.6%) were positive for *Histoplasma* urinary antigen; 551 (55.8%) had a cough, 40 (7.3%) of whom were antigen positive. Among 102 (10.3%) participants with cutaneous lesions, only 5 (4.9%) tested positive for *Histoplasma* urinary antigen. No clinical signs or symptoms distinguished tuberculosis from disseminated histoplasmosis (Table 4). Using the Xpert MTB/RIF assay, we identified 117 (11.8%) participants who tested positive for tuberculosis.

**Table 4:** Clinical features of participants in study of prevalence of histoplasmosis among persons with AIDS, Nigeria

Clinical features	No. (%) participants	No. histoplasmosis urine Ag+/total no. (%)	p value
Fever			0.602*
N	290 (29.4)	20/290 (6.9)	
Y	698 (70.6)	56/698 (8)	
Cough			0.631*
N	437 (44.2)	36/437 (8.2)	
Y	551 (55.8)	40/551 (7.3)	
Weight loss			0.882*
N	200 (20.2)	16/200 (8.0)	
Y	788 (79.8)	60/788 (7.6)	
Diarrhea			0.055*
N	733 (74.2)	49/733 (6.7)	
Y	255 (25.8)	27/255 (10.6)	
Hepatomegaly			0.722†
N	944 (95.5)	72/944 (7.6)	
Y	44 (4.5)	3/44 (6.8)	
Central nervous system symptoms			0.166†
N	875 (88.6)	71/875 (8.1)	
Y	113 (11.4)	5/113 (4.4)	
Splenomegaly			0.307†
N	955 (96.7)	75/955 (7.9)	
Y	33 (3.3)	1/33 (3.0)	
Lymphadenopathy			0.163*
N	856 (86.6)	70/856 (8.2)	
Y	132 (13.4)	6/132 (4.5)	
Cutaneous lesions			0.329*
N	886 (89.7)	71/886 (8.0)	
Y	102 (10.3)	5/102 (4.9)	
Oral mucosal lesions/ulcers			0.562†
N	920 (93.1)	72/920 (7.8)	
Y	68 (6.9)	4/68 (5.9)	
GeneXpert			0.588†
Negative	871 (88.2)	67/871 (7.7)	
Positive	117 (11.8)	9/117 (7.7)	

*Fisher exact test. †Pearson χ^2^ test.

Among participants, 420 (42.5%) had CD4 cell counts of 101–200 cells/mm^3^ (IQR 126.00–181.25 cells/mm^3^), but only 23/420 (5.5%) were *Histoplasma* urinary antigen positive; 303 (30.7%) were classified as having WHO clinical stage 3 or 4 disease, of whom 30/303 (9.9%) were antigen positive (Table 3). Despite comprising only 129/988 (13.1%), the lowest number of participants, those in the 0–50 cells/mm^3^ CD4 cell count group, had the highest (15/129, 11.6%) frequency of *Histoplasma* urinary antigen positivity (Table 3).

Eleven (1.1%) participants died during the 30-day study period, 2 from Port Harcourt and 9 from Ibadan; 2/76 (2.6%) were positive for *Histoplasma* antigenuria, 1 co-infected with *Mycobacterium tuberculosis*. The other 9 who died had negative tests for histoplasmosis and tuberculosis; cause of death was not determined in these cases.

### Histoplasmosis and Tuberculosis Coinfection

Nine (11.8%) participants, 8 female, had both histoplasmosis and tuberculosis. Co-infection occurred in all age groups. Seven of the participants were stage 3 or 4 HIV patients and 2 had 101–200/mm^3^ CD4 counts (Table 3). One co-infected participant, a 32-year-old hospitalized patient with a working diagnosis of stage 4 HIV with pulmonary tuberculosis, died during the course of the study (Figure).

**Figure:**
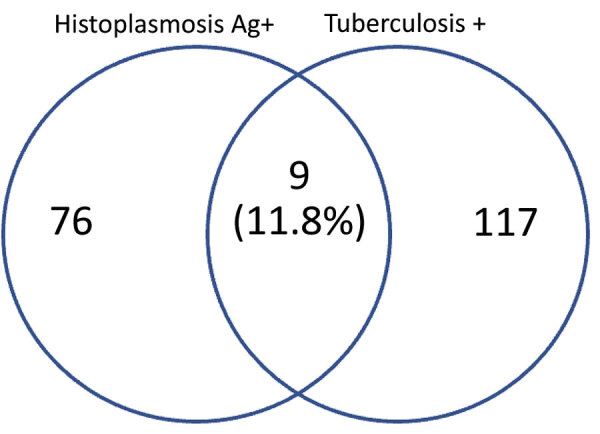
Case-patients with tuberculosis-histoplasmosis co-infection in study of prevalence of histoplasmosis among advanced HIV disease patients in Nigeria. Ag, antigen; +, positive.

## Discussion

Although histoplasmosis is endemic in Nigeria, published data have been restricted to case reports that largely predate the HIV era. Disease incidence has not been well characterized, especially among persons living with AHD (*1*). We attempted to determine the frequency of disseminated histoplasmosis in the HIV population of Nigeria and found that >7% of persons with AHD have probable histoplasmosis on the basis of European Organisation for Research and Treatment of Cancer Mycoses Study Group consensus definitions.

The countrywide prevalence of *Histoplasma* antigenuria among AHD patients in this study was <26% in Cameroon and <14% in South Africa (*8*,*17*). Whereas we used a previously validated monoclonal *Histoplasma* galactomannan enzyme immunosorbent assays to detect *Histoplasma* antigen (*18*), the study from Cameroon used a different commercial assay. It has been acknowledged that using a higher cutoff would have been more realistic and would have changed prevalence to 8%, which is closer to our findings. The lower rate in our study may have been because of technical factors such as length and conditions of storage of urine samples because specimens had to be transferred to a central location for testing to optimize the use of the antigen detection kits. Furthermore, the studies (*8*,*17*) were both conducted in single locations in South Africa and Cameroon that might both have been hyperendemic for histoplasmosis. Our multicenter study showed regional variability with prevalence ranging from 5.6% in the North Central zone to 12.9% in the South West. Even within regions, prevalence varied widely from site to site. Such variability was similarly described among regions in a multicenter study conducted in Brazil (*5*) that demonstrated a pooled prevalence of 21.6% from 14 centers, far exceeding the pooled prevalence from Nigeria. However, in the study from Brazil, use of the antigen detection method was combined with classical mycology tests including culturing, whereas we used only antigen detection (*5*). Laboratory tests for histoplasmosis are seldom performed in Nigeria because of a combination of lack of awareness, facilities, biosafety cabinets, and staff with the expertise needed to perform isolator methods of blood culturing and other laboratory testing.

Histoplasmin skin sensitivity rates predict the level of exposure to *Histoplasma* spp. in a given geographic location (*13*,*19*,*20*). Surprisingly, antigenuria prevalence did not correlate well with histoplasmin reactivity rates observed in a previous multicenter survey (*13*) that included 4 of the sites in our study: Benin City, Calabar, Ibadan, and Lagos. Benin, which recorded the highest skin sensitivity in the previous study (*13*), ended up with the lowest antigenuria prevalence in our study. It is noteworthy that in the histoplasmin sensitivity survey (*13*), skin sensitivity was significantly associated with study site. A corresponding association between site and outcome of interest, *Histoplasma* antigenuria, was not demonstrated in this study, which suggests that other factors, such as the extent of immunosuppression, may have played a greater role in determining antigenuria prevalence. The histoplasmin employed in the skin sensitivity study is known to be cross-reactive for *H. capsulatum* var. *capsulatum* and *H. capsulatum* var. *duboisii* both of which cause disseminated histoplasmosis in persons with AHD and are present in Nigeria (*19*,*20*). On the other hand, there is no evidence that the EIA deployed in this study, or any other antigen detection method for that matter, reliably detects *H. capsulatum* var. *duboisii*. In a review of histoplasmosis caused by *H. capsulatum* var. *duboisii*, diagnosis relied mostly on direct examination of body fluids and skin scrapings or histopathologic examination of clinical specimens; few were confirmed by culture or PCR and none relied on *Histoplasma* antigen detection (*21*). Because of this methodologic variability among studies, the effect on observed antigenuria prevalence of *Histoplasma* spp. distribution in the various study sites deserves further investigation.

As observed in other studies, exposure to classic environmental risk factors such as caves, heavy construction, fruit trees, and poultry were not notable risk factors for antigenuria in this study (*8*,*22*). However, contrary to findings from Cameroon, occupation was linked to positivity, with some skilled laborers, including painters, electricians, and plumbers, being more at risk than others (*8*). Another notable risk factor was smoking. Although not historically associated with progressive disseminated histoplasmosis, smoking has been recognized as a risk factor for the chronic pulmonary form of the disease (*22*).

Co-infection occurs commonly in AHD patients who have progressive disseminated histoplasmosis. Multiple studies from the Americas report tuberculosis as the most common coinfection (*23*–*27*). In the index cohort, 11.8% of participants with antigenuria had tuberculosis co-infection, which is close to the tuberculosis co-infection rate of 15.4% of participants with histoplasmosis in Brazil and 13.1% in Guatemala, both high-burden tuberculosis countries (*5*,*28*). The fact that histoplasmosis is often mistaken for and can coexist with tuberculosis is a substantial confounder in areas where the diseases are coendemic. Because tuberculosis awareness has grown and diagnostics have become more readily available, a diagnosis of tuberculosis alone might explain the signs and symptoms similar between the diseases, hiding diagnosis of the more obscure and neglected histoplasmosis in AHD patients. This shortfall suggests the need for active histoplasmosis screening in persons suspected to have tuberculosis, irrespective of confirmation with GeneXpert or other diagnostics. It is also critical to ensure that patients who screen positive for histoplasmosis can receive treatment. Several participants found to have probable histoplasmosis in this study were not treated because of financial constraints. Therefore, histoplasmosis treatment should also be included in the AHD package of care.

We found that 6.6% of participants with antigenuria had skin lesions, similar to what was found in Cameroon (6%) (*8*). However, among participants with lesions, histoplasma urinary antigen was no more common (p = 0.329). Skin lesions, which occur in 10%–25% of AIDS patients with disseminated histoplasmosis, have been linked with genetic variation among specific strains of the fungus that are dermatotropic or might be markers of histoplasmosis diagnosis when made at a very late stage (*29*). When present, biopsied lesions provide useful specimens for diagnostic confirmation of histoplasmosis. However, lesions were not very common among participants in our study, requiring us to use more available specimens. In addition, the skilled personnel needed to perform these biopsies might not be available in some settings.

One major strength of this study was that we included sites in virtually all the geopolitical zones in Nigeria that have had the most reported cases of histoplasmosis in the past. However, a study limitation was our lack of the mycology data from cultures or PCR needed to provide definitive proof of histoplasmosis and clarify the relative contributions of *H. capsulatum* var. *capsulatum* and *H. capsulatum* var. *duboisii* to its prevalence in Nigeria. Second, because it is unclear whether detecting *Histoplasma* antigen in urine provides reliable data for diagnosing *H. capsulatum* var. *duboisii*–caused histoplasmosis, we might have underestimated histoplasmosis prevalence. Third, the possibility of false positive antigenuria results cannot be entirely ruled out; however, although not tested on samples from the settings in our study, the assay we used has been validated in several studies to have good sensitivity and specificity. Fourth, our selection criteria increased the pretest probability for histoplasmosis among this cohort of participants. Fifth, we might have recorded some false-negative results as a consequence of the prolonged storage of samples. Another limitation was the lack of detailed ancillary tests, such as lactate dehydrogenase, aminotransferase, alkaline phosphatase, ferritin, and complete blood counts, which would have helped characterize patients.

Much remains to be elucidated about histoplasmosis in Nigeria, but this study confirms that it is certainly underreported among persons with HIV and AIDS, partly obscured by a diagnosis of tuberculosis, a disease with several manifestations in common with histoplasmosis. Further research using highly sensitive diagnostic approaches such as PCR and bone marrow examination is needed to gain insight into the precise epidemiology of the disease in Nigeria. To encourage proactive searching for histoplasmosis, use of specific diagnostic tools, including culturing, needs to be scaled up and management guidelines for AHD patients revised. After diagnosis, patients should be treated with appropriate antifungal agents, following the 2020 WHO guidelines. Patients suspected or confirmed to have tuberculosis should be investigated for histoplasmosis as well. Development of a molecular test in an easy-to-use format, such as the GeneXpert platform, that could be deployed in HIV treatment centers would be welcome.

In conclusion, histoplasmosis is not uncommon among AHD patients in Nigeria. Therefore, *Histoplasma* antigen screening should be included in the AHD package of care as a matter of urgent need to improve efficiency of diagnosis and reduce illness and death from histoplasmosis in an at-risk population.

Appendix. Geographic variation in histoplasmosis prevalence from study of prevalence of histoplasmosis among advanced HIV disease patients in Nigeria.
