# Supplementary material for: Prevalence of Histoplasmosis among Persons with Advanced HIV Disease, Nigeria
Source: Emerg Infect Dis. 2022 Nov;28(11):2261–9. doi: 10.3201/eid2811.220542 (PMC9622240; doi:10.3201/eid2811.220542)
Supplement: Supplementary file 1 — Appendix. Geographic variation in histoplasmosis prevalence from study of prevalence of histoplasmosis among advanced HIV disease patients in Nigeria. [file 22-0542-Techapp-s1.pdf]

Article DOI: https://doi.org/10.3201/eid2811.220542

Prevalence of Histoplasmosis among Persons with Advanced HIV Disease, Nigeria

Appendix

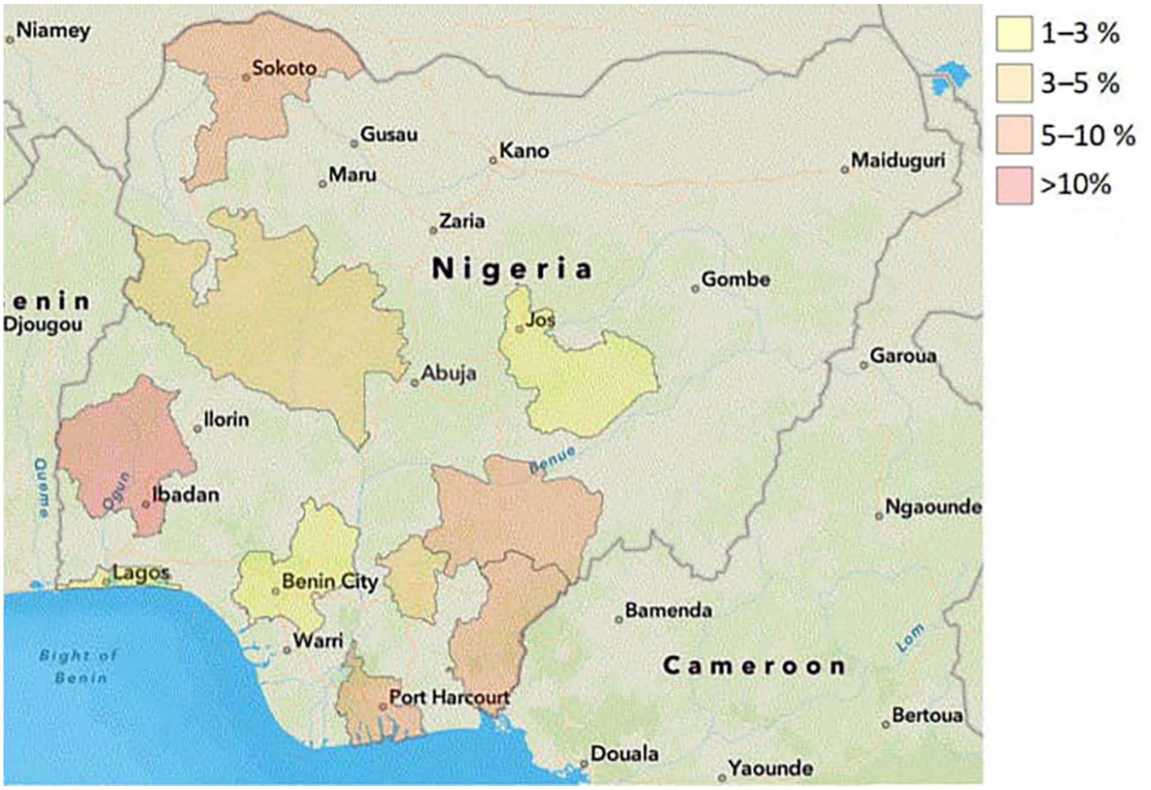

Appendix Figure. Geographic variation in histoplasmosis prevalence from study of prevalence of histoplasmosis among advanced HIV disease patients in Nigeria.
